# Endophytic fungi as sustainable tools for Fusarium head blight control in cereals: mechanisms, applications and limitations

**DOI:** 10.3389/fpls.2026.1869580

**Published:** 2026-07-31

**Authors:** Zuzana Antalová, Dominik Bleša, Marta Zavřelová, Pavel Matušinsky, Jan Šafář

**Affiliations:** 1 Institute of Experimental Botany of the Czech Academy of Sciences, Centre of Plant Structural and Functional Genomics, Olomouc, Czechia; 2 Agrotest Fyto, Ltd., Kroměříž, Czechia; 3 Department of Experimental Biology, Faculty of Science, Masaryk University, Brno, Czechia; 4 Department of Botany, Faculty of Science, Palacký University in Olomouc, Olomouc, Czechia

**Keywords:** biological control, cereals, deoxynivalenol, endophytic fungi, Fusarium head blight, induced resistance, integrated disease management, mycotoxins

## Abstract

Fusarium head blight (FHB) remains one of the most difficult cereal diseases to manage because disease severity and mycotoxin contamination are shaped by pathogen diversity, weather conditions, host susceptibility, crop residues, and the narrow timing window for fungicide application. Endophytic fungi have been proposed as complementary tools within integrated FHB management because they may suppress *Fusarium* spp. directly, reduce mycotoxin accumulation, compete for ecological niches, or prime host defence responses. This review evaluates the evidence supporting endophyte-mediated FHB control in cereals, distinguishing mechanisms demonstrated in cereal-*Fusarium* systems from those inferred from broader plant–endophyte studies. We compare application strategies including residue treatment, spike application, seed or rhizosphere inoculation, and postharvest approaches, with emphasis on their practical advantages, limitations, and current level of validation. Available studies indicate that selected fungal endophytes and endophyte-associated fungi can reduce FHB symptoms and, in some cases, deoxynivalenol accumulation under controlled or field-oriented conditions; however, field consistency, formulation, delivery, ecological safety, compatibility with existing management practices, and regulatory requirements remain major barriers to adoption. We conclude that endophytes are unlikely to replace fungicides or host resistance in the near term, but they represent promising components of integrated FHB management when supported by strain-specific evidence, quantitative disease and mycotoxin data, and multi-environment validation.

## 1 Introduction

Fusarium head blight (FHB) remains one of the most challenging diseases of cereals because its management is constrained by the biological complexity of the disease, the diversity of causal agents, the narrow window for effective intervention, and the frequent mismatch between symptom reduction and mycotoxin mitigation. FHB management currently relies on the integration of agronomic practices, partially resistant cultivars, fungicide applications, and disease monitoring. However, none of these strategies provides complete or consistently reliable control. Crop rotation and residue management can reduce inoculum pressure, but their effectiveness depends on preceding crops, tillage system, residue decomposition, and weather conditions. Moderately resistant cultivars reduce disease severity but do not provide complete resistance (Gomes Moraes et al., 2026). Fungicide-based control, particularly with triazole fungicides, remains an important component of integrated management, yet its efficacy is limited by application timing during anthesis, weather-dependent infection risk, species-specific fungicide sensitivity, and the potential selection of less sensitive *Fusarium* populations (Blandino et al., 2012; Torres et al., 2019; Tini et al., 2020; Alisaac and Mahlein, 2023). These limitations are particularly important because FHB is caused by a complex of *Fusarium* species and *Microdochium* species rather than by a single pathogen, and these taxa differ in ecology, aggressiveness, mycotoxin production, and response to fungicides (Parry et al., 1995; Bottalico and Perrone, 2002; Xu et al., 2005; Tini et al., 2020; Alisaac and Mahlein, 2023).

Biological control has therefore received increasing attention as a component of sustainable crop protection. Biocontrol uses living organisms, including microorganisms and viruses, to suppress pathogens, pests, or weeds, and is generally associated with lower environmental impact, reduced chemical input, and slower development of resistance compared with repeated pesticide use (Stenberg et al., 2021). In the context of FHB, microbial biocontrol is not expected to fully replace resistant cultivars, agronomic measures, fungicides, or forecasting systems. Rather, its value lies in complementing these approaches by reducing pathogen survival, suppressing infection, interfering with mycotoxin accumulation, or priming host defence. Such complementarity is particularly relevant for cereal production systems seeking to reduce chemical inputs while maintaining grain yield and safety.

Endophytes represent a biologically distinct and practically attractive group of microorganisms for such integrated strategies. They are commonly defined as fungi, bacteria, or actinomycetes that colonize internal plant tissues for at least part of their life cycle without causing visible disease symptoms (Stone et al., 2000). Many endophytes can enhance plant growth, improve tolerance to abiotic stress, or contribute to resistance against pathogens through direct antagonism, competition, metabolic interactions, and modulation of host defence responses (Hardoim et al., 2015; Bamisile et al., 2018; Fontana et al., 2021; Gupta et al., 2021; Byregowda et al., 2022; Li et al., 2025). They also produce diverse secondary metabolites with potential applications in medicine, industry, and agriculture (Alam et al., 2021; Jha et al., 2023; Muhammad et al., 2024). However, the term “endophyte” is often used broadly in the biocontrol literature, and not all microorganisms applied to cereals act as true internal colonizers throughout the relevant stages of host development. Some candidates may function primarily as rhizosphere colonizers, epiphytes, residue saprotrophs, antagonists, or transient endophyte-like organisms. This distinction is critical for FHB biocontrol because the biological requirements for suppressing *Fusarium* on crop residues, colonizing roots, protecting spikes during anthesis, or reducing postharvest mycotoxin risk are not equivalent.

Among endophytic microorganisms, fungal endophytes have received particular attention in cereal–*Fusarium* systems. Several fungal taxa have been reported to inhibit *Fusarium* growth, reduce FHB symptoms, decrease deoxynivalenol accumulation, or activate cereal defence responses under *in vitro*, controlled-environment, greenhouse, or field-oriented conditions. Nevertheless, the available evidence remains uneven. Many studies demonstrate strong antagonism in dual-culture assays, whereas fewer provide quantitative data on FHB severity, *Fusarium* biomass, deoxynivalenol concentration, yield-related traits, or multi-environment field performance (**Table 1**). Moreover, mechanisms described for endophyte-mediated stress tolerance in other plant systems cannot automatically be extrapolated to FHB control in cereals. A critical synthesis must therefore distinguish mechanisms experimentally demonstrated in cereal-*Fusarium* pathosystems from mechanisms inferred from broader plant-endophyte literature.

**Table 1:** Representative endophytic and endophyte-associated fungi evaluated against *Fusarium* spp. or Fusarium head blight (FHB) in cereal-related systems.

Fungus / strain	Target pathogen	Application context	System	Main effect category	Reference
*Chaetomium globosum*	*F. graminearum*	Dual culture / conidial germination assay	*In vitro*	*Fusarium* growth reduction	Xiao et al., 2013
Selected endophytic fungi	*F. graminearum* and other wheat pathogens	Antagonism screening	*In vitro*	*Fusarium* growth reduction	Abaya et al., 2021; Podgórska-Kryszczuk et al., 2022
*Trichoderma asperellum* T25	*F. graminearum*	Antagonist application / controlled assay	Controlled conditions	*Fusarium* growth reduction; plant defence induction	Pedrero-Méndez et al., 2025
*Trichoderma harzianum* T136	*F. graminearum*	Antagonist application / controlled assay	Controlled conditions	*Fusarium* growth reduction; plant defence induction	Pedrero-Méndez et al., 2025
*Trichoderma simmonsii* T137	*F. graminearum*	Antagonist application / controlled assay	Controlled conditions	*Fusarium* growth reduction; FHB symptom reduction; plant defence induction	Pedrero-Méndez et al., 2025
*Trichoderma gamsii* T6085	*F. graminearum* / *F. culmorum*	Spike-associated application	Controlled / field-oriented	FHB symptom reduction; microbiome modulation	Alukumbura et al., 2022
*Trichoderma harzianum*	*Fusarium* spp. / *Gibberella zeae*	Residue colonization	Residue / field-oriented	Inoculum reduction	Fernandez, 1992
*Trichoderma gamsii*	*Fusarium* spp.	Residue treatment	Residue / field-oriented	Inoculum reduction; *Fusarium* growth reduction	Sarrocco et al., 2019, 2021
*Trichoderma atroviride*	*Fusarium* spp.	Residue treatment	Residue / field-oriented	Inoculum reduction; *Fusarium* growth reduction	Sarrocco et al., 2019, 2021
*Microsphaeropsis* sp.	*Gibberella zeae* / *F. graminearum*	Residue treatment	Residue assay	Inoculum reduction	Bujold et al., 2001
*Clonostachys rosea*	*F. graminearum* / FHB complex	Spike spray or residue-associated application	Controlled / field-oriented	FHB symptom reduction; *Fusarium* growth reduction	Schisler et al., 2006; Xue et al., 2014
*Metarhizium anisopliae*	*F. graminearum*	Conidial suspension; seed or spike application	Controlled / greenhouse	FHB symptom reduction; DON reduction	Hao et al., 2021
*Fusarium oxysporum* non-pathogenic isolate	*F. graminearum*	Endophyte pretreatment before pathogen inoculation	Controlled / in planta	FHB symptom reduction; DON reduction; niche competition	Noel et al., 2022
*Fusarium commune*	*F. graminearum*	Endophyte pretreatment before pathogen inoculation	Controlled / in planta	FHB symptom reduction; DON reduction; niche competition	Noel et al., 2022
*Alternaria destruens*	*F. graminearum*	Endophyte pretreatment before pathogen inoculation	Controlled / in planta	FHB symptom reduction; DON reduction; niche competition	Noel et al., 2022
*Penicillium olsonii* ML37	*F. graminearum* / FHB challenge	Spike colonization / pretreatment	Controlled / transcriptomic	Plant defence induction; FHB symptom reduction	Rojas et al., 2022
*Purpureocillium lilacinum* YZ1	*F. graminearum*	Endophyte inoculation; seedling and anthesis-stage challenge	Glasshouse / field	Plant defence induction; FHB symptom reduction; colonization confirmed	Kimotho et al., 2024
*Serendipita indica*	*Fusarium* spp. / FHB	Seed, root or rhizosphere inoculation	Controlled / field-oriented	FHB symptom reduction; DON reduction; growth / yield improvement	Rabiey and Shaw, 2016
*Sarocladium zeae*	FHB pathogens	Seed-associated or plant inoculation	Controlled experiment	FHB symptom reduction; DON reduction	Kemp et al., 2020
Selected spike-applied endophytic fungi	*Fusarium* spp.	Spike application / transcriptomic evaluation	Controlled / mechanistic	Plant defence induction	Rojas et al., 2020

Effect categories: *Fusarium* growth reduction = inhibition of mycelial growth, conidial germination, sporulation, or pathogen biomass; FHB symptom reduction = reduction of disease incidence, severity, spikelet infection, bleaching, or necrosis; DON/mycotoxin reduction = reduced deoxynivalenol or other *Fusarium* mycotoxins; plant defence induction = activation or priming of host defence responses; inoculum reduction = reduced pathogen survival, perithecia formation, or ascospore production on residues; microbiome modulation = changes in spike-, root-, or residue-associated microbial communities linked to disease suppression; colonization confirmed = direct evidence of endophytic or tissue-associated colonization.

This review focuses on endophytic and endophyte-associated microorganisms, with particular emphasis on fungal endophytes, as potential components of integrated FHB management in cereals. Unlike general reviews of FHB management or plant endophytes, we aim to evaluate the evidence according to three practical questions: which microorganisms have been tested against *Fusarium* spp. or FHB in cereal systems, which mechanisms are supported by direct experimental evidence, and which application strategies appear most feasible for translation from laboratory screening to field deployment.

## 2 Review approach and conceptual boundaries

This review was prepared as a critical narrative review supported by a structured literature search. Literature searches were conducted in Web of Science Core Collection, Scopus, PubMed, and Google Scholar up to March 2026, using combinations of the following terms: Fusarium head blight, *Fusarium*, endophytic fungi, fungal endophytes, endophyte-associated fungi, biological control, biocontrol, deoxynivalenol, DON, wheat, barley, and cereals. Additional relevant studies were identified from the reference lists of selected articles. Priority was given to studies conducted in wheat, barley, and other cereal systems, and to studies reporting quantitative outcomes such as pathogen inhibition, disease severity, *Fusarium* biomass, deoxynivalenol accumulation, yield-related traits, or host defence responses. Studies from non-cereal or non-FHB systems were included only when they provided mechanistic insight relevant to endophyte-mediated protection, and these mechanisms are distinguished from those experimentally demonstrated in cereal-*Fusarium* systems. Because this was a critical narrative review rather than a systematic review, no formal systematic screening protocol, risk-of-bias assessment, or meta-analysis was performed.

Because the term “endophyte” is used inconsistently in the biocontrol literature, this review distinguishes true endophytes from endophyte-associated biological control agents. True endophytes are considered microorganisms that colonize internal plant tissues for at least part of their life cycle without causing visible disease symptoms. In contrast, some organisms discussed in the context of FHB management may act primarily as rhizosphere colonizers, spike epiphytes, residue saprotrophs, mycoparasites, or transient endophyte-like colonizers. These organisms are included when they provide relevant evidence for FHB suppression, but their ecological role and application niche are considered separately. The aim of this review is not to provide a systematic meta-analysis, but to synthesize available evidence, identify promising application routes, and define the translational gaps that currently limit the use of endophytic fungi in practical FHB management.

## 3 Fusarium head blight as a target for integrated biocontrol

Fusarium head blight is not a single-pathogen disease but a complex and dynamic pathosystem involving several *Fusarium* species and, in some regions, *Microdochium* species. The most frequently reported causal agents include *F. graminearum, F. culmorum, F. avenaceum, F. poae*, and the non-toxigenic species *Microdochium nivale* and *M. majus* (Parry et al., 1995; Xu et al., 2005; Becher et al., 2013; Karlsson et al., 2021). These species differ in ecology, aggressiveness, climatic preference, fungicide sensitivity, and mycotoxin profile, which complicates both disease prediction and disease management. In addition, several *Fusarium* species can persist on crop residues as saprotrophs, contributing to inoculum carryover between seasons, while some isolates may form asymptomatic associations with plants that resemble endophytism or weak pathogenicity (Gerlach and Nirenberg, 1982; Kistler, 1997; Leslie and Summerell, 2006; Summerell et al., 2010; Nicolaisen et al., 2014; Edel-Hermann and Lecomte, 2019). This ecological flexibility is central to the difficulty of FHB management and also explains why biological control strategies must be evaluated in relation to specific infection stages and ecological niches.

The economic and food safety relevance of FHB results not only from yield loss but also from grain contamination with mycotoxins. Severe epidemics can reduce cereal yield substantially, with losses reaching 50-70% in some regions and up to 79% in individual epidemic situations (Nganje et al., 2004; McMullen et al., 2012; Obanor et al., 2013; Palazzini et al., 2015; Ma et al., 2020). *Fusarium* species associated with FHB produce trichothecenes such as deoxynivalenol and nivalenol, as well as zearalenone and fumonisins, which compromise grain quality and pose risks to humans and livestock (Moonjely et al., 2023). European monitoring data further demonstrate that deoxynivalenol contamination is frequent and highly variable among countries and production systems, with some wheat samples exceeding legal limits for food use (Johns et al., 2022). Therefore, any biological control approach targeting FHB should be assessed not only by visible symptom reduction but also by its effect on *Fusarium* biomass, mycotoxin accumulation, and grain quality.

Current FHB management relies on the integration of agronomic practices, moderately resistant cultivars, fungicides, and monitoring systems. Crop rotation, ploughing, and residue management can reduce inoculum by limiting perithecia formation and ascospore release from infected residues (Dill-Macky and Jones, 2000; Salgado et al., 2014; Friberg et al., 2019; McKee et al., 2019; Cowger et al., 2020). However, intensive soil disturbance may conflict with reduced-tillage and soil-conservation principles, and the effectiveness of residue management depends strongly on preceding crops, weather conditions, and decomposition dynamics (Blandino et al., 2012; Matusinsky et al., 2016; Torres et al., 2019). Resistant cultivars are an essential component of FHB management, but complete resistance is not available. Instead, wheat resistance is partial and includes resistance to initial infection, spread within the spike, toxin accumulation or detoxification, kernel infection, and maintenance of grain quality (Schroeder and Christensen, 1963; Miller and Arnison, 1986; Wang and Miller, 1988; Mesterházy, 1995; Mesterházy et al., 2005; Martin et al., 2017). Several resistance loci have been identified, including *Fhb1–Fhb9*, but their effectiveness is influenced by genetic background, environment, and pathogen pressure (Zhang et al., 2004; Liu et al., 2006; Cuthbert et al., 2007; Qi et al., 2008; Xue et al., 2010, 2011; Cainong et al., 2015; Guo et al., 2015; Li et al., 2019; Su et al., 2019; Wang et al., 2020, 2024).

Chemical control remains important but also has clear constraints. Triazole fungicides such as metconazole, prothioconazole, and tebuconazole can reduce FHB severity and mycotoxin risk when applied at the appropriate timing, particularly around anthesis (Paul et al., 2008; Willyerd et al., 2012). However, the optimal treatment window is short and weather-dependent, and multiple applications may be required under sustained infection pressure (D’Angelo et al., 2014; Freije and Wise, 2015; Wegulo et al., 2015). Repeated fungicide use also increases production costs and may contribute to the selection of *Fusarium* populations with reduced sensitivity to fungicides (Anderson et al., 2020; Zhao et al., 2022). Moreover, species-specific differences in fungicide sensitivity can influence the local composition of the FHB complex, as shown for differences between *F. avenaceum* and *F. graminearum* in sensitivity to metconazole (Tini et al., 2020).

These limitations indicate that FHB is best viewed as a target for integrated disease management rather than for single-method control. Endophytic fungi and endophyte-associated biological control agents may complement existing strategies by acting at different stages of the disease cycle, including residue-borne inoculum, spike infection, host defence priming, and postharvest mycotoxin risk. Their practical value should therefore be assessed in relation to application route, timing, host genotype, environmental conditions, and their ability to reduce both disease symptoms and mycotoxin contamination.

## 4 Mechanisms of endophyte-mediated suppression of Fusarium head blight

Endophytic fungi may contribute to FHB suppression through several non-exclusive mechanisms, including direct antagonism of *Fusarium* spp., competition for ecological niches and nutrients, mycoparasitism, modulation of host defence, and interference with mycotoxin accumulation. However, the level of evidence differs substantially among mechanisms. Some effects have been demonstrated directly in cereal-*Fusarium* systems, whereas others are inferred from broader plant-endophyte studies. This distinction is important because mechanisms observed in roots, leaves, or non-cereal hosts may not necessarily operate during *Fusarium* infection of cereal spikes.

### 4.1 Direct antagonism against *Fusarium* spp.

Direct antagonism is the most frequently tested mechanism in initial screening of endophytes for FHB biocontrol. In dual-culture assays, endophytic fungi can inhibit *Fusarium* growth through antibiosis, competition for space and nutrients, volatile or diffusible metabolites, or physical interactions with pathogen hyphae. Such assays are useful for identifying candidates capable of suppressing mycelial growth or conidial germination, although they do not reproduce the complexity of host colonization, spike infection, or field environments. Several endophytic fungi have been identified through this approach. For example, Xiao et al. (2013) screened endophytic fungi isolated from *Ginkgo biloba* and identified *Chaetomium globosum* as an effective antagonist of *F. graminearum*, reducing mycelial growth and conidial germination. Similar screening studies have identified endophytes capable of inhibiting *F. graminearum* and other wheat pathogens *in vitro* (Abaya et al., 2021; Podgórska-Kryszczuk et al., 2022).

Direct antagonism may also involve mycoparasitism or interference competition. Mycoparasitic fungi can weaken pathogen hyphae through physical contact, enzymatic degradation, or nutrient extraction, whereas interference competition limits pathogen access to infection sites and resources. These mechanisms are well described for several biological control agents, including *Trichoderma* spp. and *Clonostachys rosea*, but their relevance to FHB depends on the ecological context in which the antagonist is applied. For example, organisms applied to crop residues may reduce survival and sporulation of *Fusarium* before infection occurs, whereas organisms applied to spikes must persist on or within floral tissues during the narrow anthesis infection window. Therefore, direct antagonism should be interpreted in relation to the intended application strategy rather than as a universal predictor of FHB control.

### 4.2 Competition and niche occupation

Competition for ecological niches is particularly relevant in the FHB pathosystem because *Fusarium* spp. occupy multiple habitats during their life cycle, including crop residues, soil, roots, stems, and flowering spikes. Endophytic or endophyte-associated fungi may reduce disease risk by occupying infection courts, consuming available nutrients, or reducing the establishment of *Fusarium* on residues or host tissues. This mechanism is especially relevant for residue treatments and spike applications. Saprotrophic and antagonistic fungi such as *Trichoderma harzianum*, *Trichoderma gamsii*, *Trichoderma atroviride*, *Microsphaeropsis* sp., and *Clonostachys rosea* have been reported to reduce pathogen survival or inoculum production on crop debris through competition for space and nutrients (Fernandez, 1992; Bujold et al., 2001; Naef et al., 2006; Palazzini et al., 2013; Sarrocco et al., 2019, 2021). Although such organisms may not always function as true endophytes, they are relevant to integrated FHB management because they target the saprotrophic stage of *Fusarium* and may reduce primary inoculum while maintaining reduced-tillage systems.

In planta niche occupation is more difficult to demonstrate than *in vitro* antagonism. For an endophyte to contribute to spike protection, it must colonize the relevant tissues before or during pathogen infection, persist under field conditions, and avoid negative effects on host development. The “cry for help” hypothesis suggests that plants may recruit beneficial microorganisms through root exudates or nutrient leakage from aerial tissues, thereby shaping a health-promoting microbiome under stress (Haas and Défago, 2005; Bakker et al., 2018). However, in the context of FHB, the key question is not only whether an endophyte can colonize the plant, but whether its colonization coincides spatially and temporally with *Fusarium* infection and mycotoxin production. Future studies should therefore report colonization dynamics, tissue specificity, and persistence in addition to disease outcomes.

### 4.3 Activation and priming of host defence responses

Endophytes may also suppress FHB indirectly by priming or activating host defence responses. In general, endophyte-associated resistance can involve structural reinforcement of plant tissues, accumulation of phenolic compounds, callose or lignin deposition, activation of pathogenesis-related proteins, antioxidant enzyme activity, and modulation of hormone-dependent immune signalling (Benhamou et al., 1998, 2000; Vallad and Goodman, 2004; Pieterse et al., 2014; Kumar and Verma, 2018; Fontana et al., 2021; Lastochkina et al., 2021; Vlot et al., 2021; Chancellor et al., 2024). Induced systemic resistance is usually associated with jasmonic acid and ethylene signalling, whereas systemic acquired resistance is more closely related to salicylic acid signalling. Endophytes may influence both pathways, suggesting that their effects on host defence can involve crosstalk among immune signalling networks.

In cereal–*Fusarium* systems, defence activation is particularly relevant because FHB resistance depends not only on the prevention of initial infection but also on restriction of pathogen spread within the spike, detoxification or reduced accumulation of deoxynivalenol, and maintenance of grain quality. Endophyte-mediated defence responses may therefore contribute to several resistance components, including reduced infection, slower pathogen spread, enhanced cell-wall reinforcement, and altered expression of defence-related enzymes. Studies reporting activation of plant defence gene expression after endophyte application to wheat spikes provide direct support for this mechanism in the FHB context (Rojas et al., 2022; Risoli et al., 2023). However, many defence mechanisms discussed in the broader endophyte literature remain insufficiently validated in cereal spikes during *Fusarium* infection. For this reason, mechanisms such as salicylic acid-, jasmonic acid-, ethylene-, or ROS-related responses should be interpreted as experimentally demonstrated for FHB only when supported by cereal-*Fusarium* data.

Pathogenesis-related proteins and defence enzymes represent another plausible route of endophyte-mediated protection. Chitinases and β-1,3-glucanases can degrade fungal cell wall components and inhibit pathogen proliferation, while enzymes such as superoxide dismutase, peroxidase, polyphenol oxidase, and phenylalanine ammonia-lyase are frequently associated with enhanced defence and oxidative stress management (Govinda Rajulu et al., 2011; Singh and Gaur, 2017; Malto et al., 2019; Padder et al., 2022; Jha et al., 2023; Eichfeld et al., 2024). Nevertheless, the relevance of these responses to FHB control depends on their timing, tissue localization, and association with reduced *Fusarium* biomass or mycotoxin accumulation. Defence activation alone should therefore not be considered sufficient evidence of FHB biocontrol unless linked to disease or toxin outcomes.

### 4.4 Interference with deoxynivalenol accumulation

Reduction of deoxynivalenol is a central objective of FHB management because visual symptom suppression does not always correspond to lower mycotoxin contamination. Endophytic fungi may influence deoxynivalenol accumulation indirectly by reducing *Fusarium* infection, limiting fungal biomass, restricting pathogen spread, or altering host defence responses that affect toxin production. In some cases, they may also affect mycotoxin metabolism or detoxification, although direct evidence for detoxification by endophytic fungi in cereal-FHB systems remains limited.

Several studies indicate that endophyte-based treatments can reduce both FHB symptoms and deoxynivalenol accumulation. For example, *Serendipita indica* application reduced FHB disease severity and incidence and decreased deoxynivalenol concentration in winter and spring wheat samples, while also improving biomass and yield-related traits (Rabiey and Shaw, 2016). Seed coating with *Metarhizium anisopliae* and inoculation with *Sarocladium zeae* have also been associated with lower FHB symptoms and reduced deoxynivalenol levels in wheat (Kemp et al., 2020; Hao et al., 2021). These examples suggest that endophyte-mediated FHB control should be evaluated using both disease and toxin endpoints. Future studies should report deoxynivalenol concentration alongside FHB incidence or severity, grain yield, and grain quality parameters, because reduction of visible symptoms without toxin mitigation may have limited practical value.

### 4.5 Endophyte-mediated resistance and mechanisms inferred from non-FHB systems

Endophyte-mediated resistance has been described most clearly in relation to non-pathogenic or weakly pathogenic *Fusarium oxysporum* endophytes, particularly in root systems. This response appears to function partly independently of salicylic acid and jasmonic acid/ethylene signalling pathways and may involve localized cell death or alternative immune pathways (He et al., 2002; Alabouvette et al., 2009; Pieterse et al., 2014; Constantin et al., 2019; de Lamo and Takken, 2020; de Lamo et al., 2021). Although this concept is relevant for understanding how endophytes can alter host susceptibility, its direct relevance to FHB control in cereal spikes remains to be fully established. It should therefore be considered a promising mechanistic framework rather than a mechanism already broadly demonstrated for cereal–*Fusarium* head blight interactions.

Similarly, many endophytes improve plant performance under abiotic stress by modulating redox balance, nutrient uptake, phytohormone signalling, antioxidant systems, or stress-responsive gene expression (Hardoim et al., 2015; Gupta et al., 2021; Byregowda et al., 2022; Li et al., 2025). These effects may indirectly influence disease outcomes by improving host vigour or stress tolerance, but they should not be presented as direct evidence for FHB suppression unless tested under *Fusarium* challenge in cereals. Claims related to salinity, metal toxicity, heat stress, or bioremediation are therefore best treated as broader examples of endophyte functional diversity rather than as core mechanisms of FHB control. In the FHB context, the strongest mechanistic claims should be reserved for studies linking endophyte colonization or application to reduced *Fusarium* growth, reduced disease severity, altered host defence during *Fusarium* challenge, decreased deoxynivalenol accumulation, or improved grain outcomes.

To provide a structured overview of the available evidence, **Table 1** summarizes representative fungal endophytes and endophyte-associated fungi evaluated against *Fusarium* spp. or FHB in cereal-related systems. The table includes organisms acting through different ecological routes, including internal colonization, spike association, residue colonization, rhizosphere association, direct antagonism, and defence induction. Because not all candidates function as true endophytes under all experimental conditions, their role should be interpreted according to the reported application context. The table is intended as a comparative synthesis of the most relevant experimental evidence rather than as a systematic database of all microbial biocontrol studies.

## 5 Application strategies and practical feasibility: from screening to deployment

The development of endophytic fungi as biological control agents against FHB requires a stepwise evaluation process. Initial *in vitro* screening can identify candidates with direct antagonistic activity against *Fusarium* spp., but practical relevance depends on whether these organisms can colonize the appropriate host tissue, persist under variable environmental conditions, reduce disease or mycotoxin accumulation, and remain compatible with existing crop management practices. Therefore, application strategy is not a secondary technical detail but a major determinant of biocontrol performance. Endophytes or endophyte-associated fungi may target different stages of the FHB cycle, including pathogen survival on residues, infection of flowering spikes, host defence priming through seed or root colonization, or postharvest reduction of fungal growth and toxin risk (**Table 2**).

**Table 2:** Practical comparison of application strategies for endophyte-based or endophyte-associated Fusarium head blight (FHB) management.

Strategy	Target stage	Main rationale	Advantages	Main limitations	Practical readiness
Residue treatment	Saprotrophic survival / inoculum	Reduce perithecia, ascospores, residue-borne inoculum	Compatible with reduced tillage; targets primary inoculum	Weather-dependent; not necessarily true endophytism	Medium
Spike application	Anthesis infection	Direct protection of infection court	Directly relevant to FHB	Narrow timing window; formulation stress	Medium
Seed/rhizosphere application	Early colonization / priming	Establish endophyte before infection	Scalable; compatible with seed treatment	Uncertain persistence to anthesis; genotype/environment effects	Promising but variable
Postharvest treatment	Grain storage / toxin risk	Reduce fungal growth or toxin impact after harvest	Relevant to grain/feed safety	Not direct field FHB control	Peripheral/complementary

### 5.1 *In vitro* and detached-tissue screening

Dual-culture plate assays remain the most widely used first step for identifying endophytes with antagonistic activity against *Fusarium* spp. These assays can reveal inhibition of mycelial growth, conidial germination, or direct interactions between the candidate endophyte and pathogen. However, *in vitro* antagonism alone is insufficient to predict FHB suppression in planta because it does not account for host colonization, tissue specificity, environmental variation, or indirect defence-mediated mechanisms.

Detached spikelet and controlled-environment assays provide an intermediate step between plate screening and field validation. These systems allow researchers to assess whether candidate endophytes can reduce *Fusarium* infection in host tissues and may reveal candidates that would be missed by standard dual-culture assays. Rojas et al. (2020), for example, demonstrated the value of detached spikelet methods for identifying effective endophytes beyond simple plate-based antagonism. Such assays are particularly useful for ranking candidates before greenhouse or field-oriented studies, but their results should still be interpreted as preliminary unless supported by quantitative disease and mycotoxin data.

### 5.2 Application to crop residues

Residue treatment targets the saprotrophic phase of the FHB cycle, when *Fusarium* spp. survive and reproduce on infected crop debris before the next cereal crop becomes susceptible. This strategy is particularly relevant in reduced-tillage systems, where residues remain on the soil surface and can serve as sources of perithecia and ascospores. Saprotrophic or antagonistic fungi such as *Trichoderma harzianum, Microsphaeropsis* sp., *Trichoderma gamsii*, *Trichoderma atroviride*, and *Clonostachys rosea* have been reported to reduce pathogen survival or inoculum production through competition for space and nutrients or direct antagonism (Fernandez, 1992; Bujold et al., 2001; Naef et al., 2006; Palazzini et al., 2013; Sarrocco et al., 2019, 2021).

The main advantage of residue treatment is that it targets the saprotrophic stage of the pathogen and may complement conservation agriculture by reducing inoculum without requiring intensive soil disturbance. However, this approach may not involve true endophytic colonization of the crop and should therefore be considered an endophyte-associated or biological control strategy rather than a strict endophyte-based intervention. Its performance also depends on residue moisture, temperature, microbial competition, timing of application, and the capacity of the introduced organism to establish before *Fusarium* sporulation.

### 5.3 Direct application to wheat spikes

Direct application of antagonistic or endophyte-associated fungi to wheat spikes targets the infection court most relevant to FHB development. Because *Fusarium* infection occurs mainly around anthesis, spike application is conceptually close to fungicide-based FHB control and may directly reduce infection, pathogen growth, or mycotoxin accumulation. Spraying conidial suspensions of candidate fungi such as *Metarhizium anisopliae, Clonostachys rosea, F. oxysporum, F. commune*, and *Alternaria destruens* has been reported to reduce FHB symptoms or mycotoxin levels under experimental conditions (Schisler et al., 2006; Xue et al., 2014; Hao et al., 2021; Noel et al., 2022). Transcriptomic studies also indicate that spike-applied endophytes can activate defence-related gene expression in wheat, supporting a role for host response modulation in addition to direct antagonism (Rojas et al., 2022; Risoli et al., 2023; Kimotho et al., 2024).

The practical strength of spike application is its direct relevance to the infection site, but this strategy also faces the same major constraint as fungicide-based FHB control: the treatment window around anthesis is narrow and strongly weather-dependent. In addition, the wheat spike is a short-lived and environmentally exposed habitat, so successful biological control depends not only on antagonistic activity but also on the capacity of the applied fungus to establish on or within spike tissues, tolerate UV radiation, desiccation, rainfall, and fluctuating humidity, and remain active during the period of highest *Fusarium* infection risk. Recent spike-focused studies indicate that some fungal biocontrol agents (BCAs), including selected *Trichoderma* spp. and *Penicillium olsonii* ML37, may act through direct antagonism, modulation of wheat defence responses, or stabilization of the spike-associated microbiome. These findings support the view that spike application should be evaluated as an ecological intervention in the spicosphere rather than as a simple replacement for fungicide spraying. Practical deployment will therefore require optimized formulations, precise timing, validation under realistic anthesis conditions, and compatibility with existing FHB warning models, fungicide schedules, or low-input systems where chemical control is restricted.

### 5.4 Seed and rhizosphere application

Seed coating, root inoculation, and soil or rhizosphere application aim to establish endophytes early in plant development and prime host defence before *Fusarium* infection occurs. This strategy has practical appeal because seed treatment is technically simple, scalable, and compatible with existing agricultural workflows*. Serendipita indica* is one of the most studied examples in this context. Its application reduced FHB disease severity and incidence and decreased deoxynivalenol concentration in winter and spring wheat samples, while also improving biomass and yield-related traits (Rabiey and Shaw, 2016). Similarly, seed coating with *Metarhizium anisopliae* or inoculation with *Sarocladium zeae* has been associated with reduced FHB symptoms and lower deoxynivalenol levels in wheat (Kemp et al., 2020; Hao et al., 2021).

The major advantage of seed or rhizosphere application is that it may provide early and systemic benefits, including improved plant vigour, altered root or shoot physiology, and primed defence responses. However, its relevance to FHB depends on whether root or seed-associated colonization can influence susceptibility of spikes at anthesis. This requires evidence for persistence, systemic signalling, or measurable reduction of *Fusarium* infection and mycotoxin accumulation at later growth stages. Therefore, future studies should report colonization dynamics, plant developmental stage, disease outcomes, and deoxynivalenol levels rather than relying only on early growth or root colonization data.

### 5.5 Postharvest and downstream feed-related approaches

Postharvest treatment is more peripheral to field FHB control but remains relevant to grain safety because *Fusarium* spp. can continue to grow or persist on harvested grain when storage conditions are unsuitable. Microbial or biological approaches applied after harvest should therefore be clearly distinguished from endophyte-mediated disease suppression in living cereal plants. In this context, postharvest grain treatment would refer to interventions aimed at limiting fungal growth or toxin risk during storage, whereas downstream feed detoxification targets mycotoxins after contaminated grain has already entered the feed chain.

Biomin^®^ BBSH 797 falls into the latter category rather than the direct treatment of harvested grain. It is authorized in the European Union for detoxifying deoxynivalenol in the animal gastrointestinal tract by converting it to deepoxy-deoxynivalenol (Díaz-Urbano et al., 2023).

Therefore, it should be considered a downstream feed-detoxification strategy, not a field or postharvest grain treatment for FHB. This example is relevant only to the broader disease-toxin continuum and illustrates that mycotoxin risk can also be addressed after harvest, although such approaches do not reduce FHB infection in the crop.

For this reason, postharvest and feed-related approaches are best considered complementary to FHB management rather than direct substitutes for field disease control. The primary role of endophytic fungi in FHB management remains linked to preharvest processes, including inoculum reduction, spike protection, host defence priming, and reduction of disease and mycotoxin accumulation before grain enters storage or feed use.

## 6 Translational limitations and challenges for field adoption

Despite promising experimental results, several constraints currently limit the widespread use of endophytic fungi as practical tools for FHB management. The main challenge is not only the identification of antagonistic strains, but the translation of strain-specific effects into consistent, safe, scalable, and economically viable products. Many candidate endophytes perform well *in vitro* or under controlled conditions, but their efficacy often decreases or becomes variable under field conditions (O’Callaghan, 2016; Bamisile et al., 2021). These limitations are particularly relevant for FHB, where infection risk and control efficacy are strongly shaped by weather conditions during the narrow anthesis window (D’Angelo et al., 2014; Freije and Wise, 2015; Wegulo et al., 2015). Therefore, field performance should be treated as the central criterion for practical relevance.

### 6.1 Field consistency and environmental dependence

FHB development is strongly influenced by weather conditions around anthesis, and the same is likely true for endophyte-based control. A candidate strain that suppresses *Fusarium* growth under laboratory conditions may fail to colonize spikes, survive on plant surfaces, or activate host defence under dry, hot, rainy, or highly competitive field conditions. This lab-to-field gap is a recurring limitation in microbial biocontrol, where efficacy is affected by environmental fluctuation, microbial competition, host genotype, and application timing (O’Callaghan, 2016; Bamisile et al., 2021). In FHB specifically, the narrow and weather-dependent infection window around anthesis further complicates delivery, as also observed for fungicide-based management (D’Angelo et al., 2014; Freije and Wise, 2015; Wegulo et al., 2015). In addition, differences among *Fusarium* species in ecology, aggressiveness, and fungicide sensitivity may influence the outcome of integrated management strategies, including biological control (Tini et al., 2020; Alisaac and Mahlein, 2023). Therefore, promising candidates should be tested across cultivars, environments, pathogen populations, and seasons before being considered suitable for practical deployment. Single-location or single-season experiments provide useful proof of concept but are insufficient for recommending broad agricultural use.

Another source of field variability is the multi-species nature of the FHB complex. Many screening assays (**Table 1**) evaluate biocontrol candidates against a single isolate of *F. graminearum*, but field epidemics often involve co-occurring *Fusarium* species with different ecological roles, aggressiveness, mycotoxin profiles, and sensitivity to biological or chemical control. Species such as *F. culmorum, F. poae, F. avenaceum*, and members of the *F. graminearum* species complex may alter the infection environment, compete for spike niches, or influence the performance of applied biological control agents. Therefore, future screening should include regionally relevant *Fusarium* species or mixed-species challenge assays where appropriate. This would provide a more realistic assessment of whether candidate endophytes can function under the pathogen-community conditions encountered in cereal fields.

### 6.2 Formulation, delivery, and shelf life

The development of a successful endophyte-based product requires a stable formulation that maintains viability, infectivity, and efficacy during storage, transport, and field application. Formulation challenges differ among application strategies. Seed coatings require compatibility with seed handling, germination, and existing seed treatment products. Spike sprays require survival of propagules under UV exposure, desiccation, rainfall, and variable canopy microclimates. Residue applications require establishment on senescent plant material and competition with native saprotrophs. These requirements may involve different propagule types, carriers, additives, or protectants. Such constraints are common in microbial biocontrol development, where biological efficacy must be translated into stable, deliverable, and farmer-compatible products (O’Callaghan, 2016). Shelf life is particularly important for commercialization because farmers and distributors require products with predictable performance over time. However, formulation and storage stability are often underreported in experimental studies, creating a gap between biological efficacy and product readiness (Mastan et al., 2019).

### 6.3 Compatibility with integrated FHB management

Endophytic fungi are unlikely to replace resistant cultivars, agronomic practices, fungicides, or disease forecasting in the near term. Their most realistic role is as complementary components of integrated disease management. Therefore, future studies should evaluate compatibility with existing FHB strategies. This includes interactions with partially resistant cultivars, fungicide programs, crop rotation, tillage systems, and disease warning models. Resistant cultivars can substantially reduce FHB severity, but available resistance remains partial and is influenced by genetic background, pathogen pressure, and environment (Mesterházy et al., 2005; Gomes Moraes et al., 2026; Ma et al., 2025). Similarly, fungicides remain important for FHB suppression, but their efficacy depends strongly on timing around anthesis and may be affected by repeated applications or shifts in pathogen sensitivity (D’Angelo et al., 2014; Freije and Wise, 2015; Anderson et al., 2020; Zhao et al., 2022). Compatibility with fungicides is especially important because many endophyte-based approaches may be applied near the same developmental window as chemical FHB control. Some fungicides could inhibit the introduced endophyte, while compatible programs may allow reduced chemical input or improved consistency of disease and mycotoxin control. Testing endophytes only as stand-alone treatments may therefore underestimate their practical value in integrated systems.

### 6.4 Disease suppression versus mycotoxin reduction

A major limitation of many biocontrol studies is that they report visible disease reduction but do not quantify deoxynivalenol or other mycotoxins. This is problematic because FHB management is ultimately a grain safety issue as well as a disease control issue. *Fusarium* species associated with FHB produce several mycotoxins, including trichothecenes such as deoxynivalenol and nivalenol, as well as zearalenone and fumonisins, which compromise grain quality and pose risks to humans and livestock (Moonjely et al., 2023). Monitoring studies further show that deoxynivalenol contamination is frequent and highly variable among regions and production systems, with some samples exceeding legal limits for food or feed use (Johns et al., 2022; Niaz et al., 2025). Reduced symptoms do not necessarily guarantee reduced toxin contamination. Studies that demonstrate both symptom reduction and mycotoxin mitigation should be considered stronger evidence than studies based only on *in vitro* pathogen inhibition or visual disease scores. Examples such as *Serendipita indica, Metarhizium anisopliae*, and *Sarocladium zeae* indicate that some endophyte-based treatments can reduce FHB symptoms and deoxynivalenol accumulation, but these outcomes require broader validation across environments and cereal genotypes (Rabiey and Shaw, 2016; Kemp et al., 2020; Hao et al., 2021).

### 6.5 Biosafety and non-target effects

The ecological safety of endophytic fungi must be carefully evaluated before large-scale application. Some endophytes may have broad host ranges, produce bioactive metabolites, interact with native microbial communities, or shift between mutualistic, saprotrophic, and pathogenic lifestyles depending on host or environmental context. These concerns are especially relevant for fungi with broad ecological plasticity or close relationships to known plant pathogens. Additional risks include phytotoxicity, effects on non-target fungi or beneficial microorganisms, and mycoparasitism of organisms that contribute positively to soil or plant health (Fravel, 1988; Lumsden et al., 1992; Rousseau et al., 1996; Bamisile et al., 2021; Pozo et al., 2024). This issue is particularly important for *Fusarium*-related endophytes, because the boundary between pathogenic, weakly pathogenic, asymptomatic, and beneficial interactions can be context-dependent (Nicolaisen et al., 2014; Edel-Hermann and Lecomte, 2019). Safety assessment should therefore include host range, toxin production, genome-based risk indicators where available, non-target effects, and stability of the beneficial phenotype.

### 6.6 Regulatory and economic barriers

**Figure 1:**
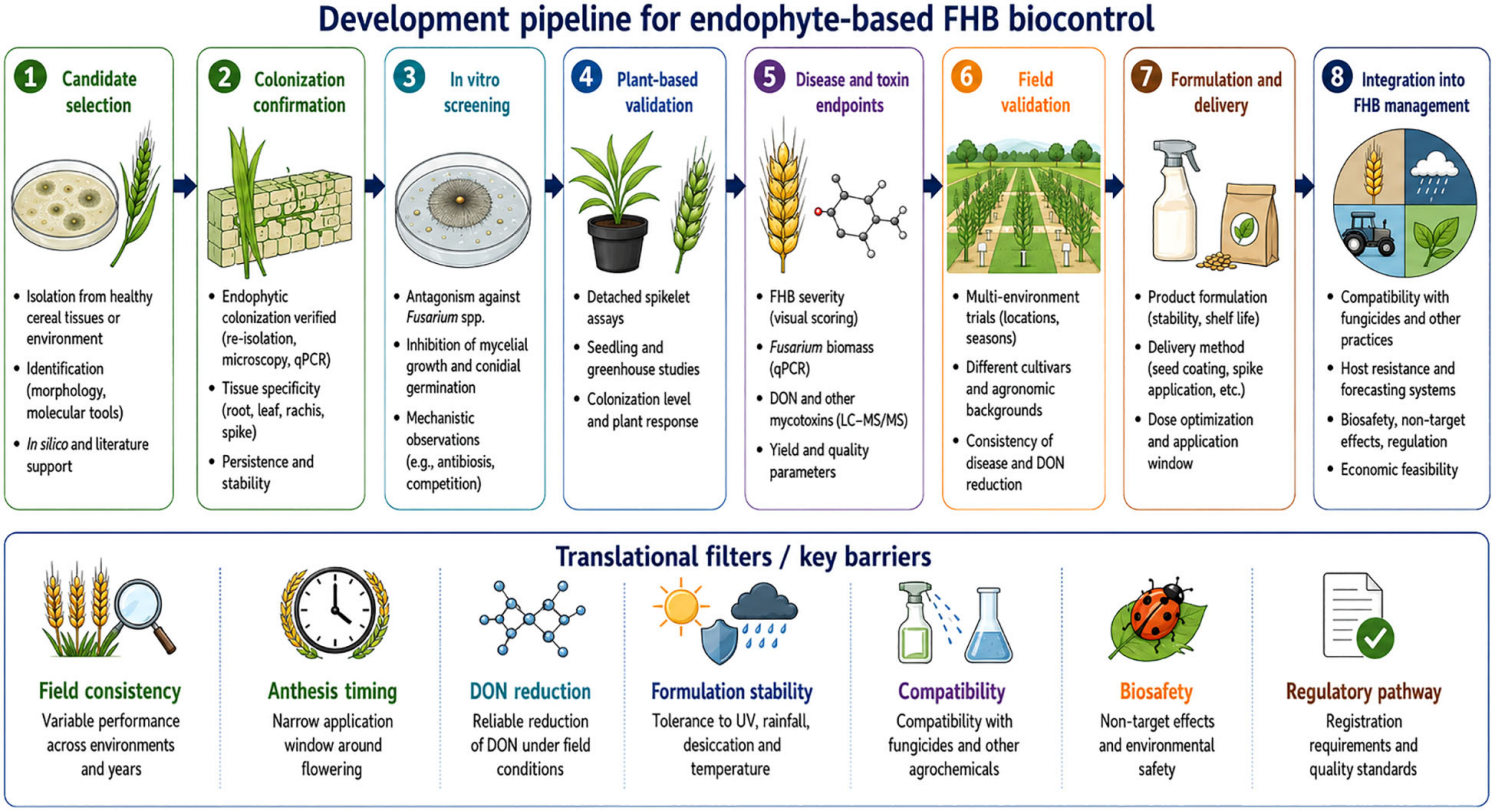
Development pipeline for endophyte-based Fusarium head blight (FHB) biocontrol. Candidate fungal endophytes should progress from isolation and identification through confirmation of endophytic colonization, *in vitro* and plant-based screening, disease and mycotoxin assessment, field validation, formulation development, biosafety evaluation, and integration into existing FHB management systems. The figure emphasizes that promising laboratory effects are not sufficient for practical use unless they are supported by quantitative disease and deoxynivalenol data, field consistency, stable delivery, and compatibility with integrated disease management.

Commercial development of microbial biocontrol products is costly and time-consuming. Candidate strains must be identified, characterized, formulated, tested for efficacy, evaluated for safety, produced at scale, and approved under relevant regulatory frameworks (Sundh and Eilenberg, 2021). These steps are particularly demanding for living microbial products because product performance depends not only on the active organism, but also on formulation, storage stability, delivery method, environmental conditions, and compatibility with existing agricultural practices (Bamisile et al., 2021). Even after registration, adoption depends on cost, ease of use, compatibility with farm operations, and consistency relative to existing fungicide-based programs. These barriers are particularly high for FHB because farmers require reliable control during a narrow infection window and because grain buyers are concerned with mycotoxin thresholds. For endophyte-based products to become practical, future research must therefore move beyond proof-of-concept screening and generate data relevant to product development, including formulation stability, dose-response relationships, application timing, field repeatability, mycotoxin mitigation, and economic feasibility (Sundh and Goettel, 2013; Sundh and Eilenberg, 2021; **Figure 1**).

The main translational gap in endophyte-based FHB control lies between promising experimental effects and reliable field performance. The strongest future candidates will be those that combine strain-specific efficacy, confirmed colonization or ecological function, reduction of both disease symptoms and deoxynivalenol, compatibility with integrated management, environmental safety, and practical formulation potential.

## 7 Conclusion and research priorities

Endophytic fungi represent promising complementary tools for the management of Fusarium head blight in cereals, but their current role should be viewed within integrated disease management rather than as replacements for resistant cultivars, agronomic measures, fungicides, or forecasting systems. Available studies show that selected fungal endophytes and endophyte-associated biological control agents can inhibit *Fusarium* spp., reduce FHB symptoms, activate host defence responses, and in some cases decrease deoxynivalenol accumulation. However, the strength of evidence varies considerably among organisms, mechanisms, and application strategies. *In vitro* antagonism is useful for candidate screening, but it is not sufficient to predict field performance or mycotoxin mitigation.

Future research should prioritize five areas. First, studies should clearly distinguish true endophytes from rhizosphere colonizers, epiphytes, residue saprotrophs, and other endophyte-associated biocontrol agents. Second, mechanisms should be linked directly to cereal-*Fusarium* systems rather than inferred uncritically from unrelated plant-endophyte interactions. Third, disease assessment should include deoxynivalenol and other mycotoxin endpoints in addition to visible FHB symptoms. Fourth, promising strains should be evaluated across host genotypes, environments, pathogen populations, and seasons. Fifth, formulation, shelf life, delivery method, compatibility with fungicides and resistant cultivars, biosafety, regulation, and economic feasibility must be addressed early in the development pipeline.

The most realistic near-term use of endophytic fungi is therefore as part of integrated FHB management, where they may reduce inoculum, complement spike protection, prime host defence, or contribute to mycotoxin risk reduction. Their successful implementation will depend on moving from descriptive screening studies toward quantitative, mechanism-informed, and field-validated research that connects biological efficacy with practical agricultural deployment.
